# “I did it”: a phenomenological study of women’s experiences of midwife-supported vaginal birth after cesarean sections in South Korea

**DOI:** 10.4069/whn.2026.08.26

**Published:** 2026-09-29

**Authors:** Uri Bang, Hyun Kyoung Kim

**Affiliations:** Department of Nursing, Kongju National University, Gongju, Korea

**Keywords:** Midwifery, Natural childbirth, Postpartum period, Qualitative research, Vaginal birth after cesarean

## Abstract

**Purpose:**

This study explored the meaning of women’s choices regarding mode of birth and the birth experiences of women in the Republic of Korea who had a vaginal birth after cesarean (VBAC) within the previous 3 months.

**Methods:**

This descriptive phenomenological study used Colaizzi’s framework. Nine postpartum women who had experienced midwife-supported VBAC at three obstetric hospitals in Seoul were recruited through purposive sampling. Data were collected through in-depth interviews from March 1, 2025, to May 7, 2026. Each participant completed a videoconference interview, and two participants completed an additional telephone interview. Data were analyzed using Colaizzi’s seven-step method, including extraction of significant statements, formulation of meanings, organization into themes, and member checking of the essential structure.

**Results:**

Three categories and 12 theme clusters were identified: “Trauma and lasting impressions of cesarean delivery,” “Motives and barriers affecting VBAC decisions,” and “Supportive relationships and transformation of the birth experience.” Respect for autonomy, a positive birth environment, and family support helped transform birth from an experience of alienation to one of participation. The statement “I did it” expressed a sense of accomplishment and recovery of the agency participants felt they had lost during previous births.

**Conclusion:**

This study suggests that, for these participants, VBAC involved more than a choice of mode of birth; it was experienced as a process of rewriting the birth narrative and recovering agency and self-efficacy. These findings may inform midwives and nurses seeking to understand and support the psychosocial experiences of women pursuing VBAC.

## Introduction

Vaginal birth after cesarean (VBAC) refers to vaginal birth in a subsequent pregnancy after a previous cesarean delivery [1]. VBAC has been proposed as an important alternative that may reduce complications associated with repeat cesarean delivery and facilitate maternal recovery [2,3]. Although advances in electronic fetal monitoring and ultrasonography have improved identification of high-risk situations during labor, they have also been accompanied by an increased tendency to perform cesarean delivery [2,4]. In the United States, the cesarean delivery rate rose from approximately 5% in the 1970s to 32.4% in 2023 [5]. In the Republic of Korea, cesarean deliveries accounted for 67.4% of births in 2024 [6]. This rate exceeds the cited World Health Organization threshold of 10% and is among the highest in countries belonging to the Organisation for Economic Co-operation and Development [7,8].

From a maternal perspective, cesarean delivery carries greater risks than vaginal birth, including higher risks of hemorrhage, infection, pain, and delayed recovery. Repeat cesarean deliveries further increase the risk of serious complications, including placenta previa and placenta accreta [9]. For women planning future pregnancies, VBAC therefore represents an important option. Compared with repeat cesarean delivery, VBAC has established benefits, including lower maternal mortality, shorter hospital stays, and a higher likelihood of breastfeeding, although it also carries a risk of maternal complications, including uterine rupture [10]. However, because the absolute incidence of uterine rupture is low, VBAC can be a reasonable and safe option for most appropriately selected women [9]. Therefore, healthcare systems should support an environment in which women at low risk for uterine rupture receive sufficient information and can make autonomous decisions about their mode of delivery [11].

National VBAC policies vary according to healthcare systems, legal frameworks, clinical guidelines, and cultures surrounding childbirth. In the United Kingdom, the National Institute for Health and Care Excellence states that VBAC is a safe option for most women with a previous cesarean delivery, and a cohort study reported that approximately 52% of eligible women attempted VBAC [12]. In the United States, the rate of attempted VBAC was 13.3% in 2018 [5]. The American College of Obstetricians and Gynecologists (ACOG) states that VBAC is a safe and appropriate option for many women with a previous cesarean delivery and reports that 60% to 80% of women who attempt VBAC have a successful vaginal birth [2]. In contrast, in South Korea, VBAC accounted for only 0.17% of all deliveries in 2024 [6].

For women, the decision to pursue VBAC is not simply a medical choice but a complex process shaped by psychological, emotional, and social factors. According to meta-syntheses of qualitative studies, women who experienced loss of control, insufficient support, or prolonged recovery after a previous cesarean delivery are more likely to view VBAC as an opportunity to reclaim their birth experience [13,14]. Women’s decisions about VBAC are also influenced by healthcare providers’ attitudes and the way information is provided, as well as by support from spouses and family members and by previous birth experiences [15,16]. However, women often make these decisions without sufficient evidence-based information, leaving them to “grope through the fog” [14].

Natural birth is a childbirth approach that minimizes medical intervention and emphasizes women’s physical autonomy and active participation, with midwives providing the central professional support [4]. Midwife-led continuity of care provides sustained, individualized support to mothers, reduces unnecessary medical interventions, and improves birth satisfaction and mother–infant attachment [17]. Support from midwives is particularly important for women attempting VBAC because continuous midwifery care contributes to higher VBAC success rates and more positive birth experiences [18]. Nevertheless, in the South Korean healthcare setting, the shortage of healthcare professionals and institutions that support VBAC structurally limits women’s choices in childbirth.

Despite the increasing recognition of the need for a supportive VBAC environment, research on VBAC remains limited. In South Korea, in-depth studies exploring the subjective experiences of women who undergo VBAC through natural birth in collaboration with midwives are particularly scarce. Existing VBAC studies have focused primarily on clinical outcomes, such as success rates, uterine rupture risk, and neonatal outcomes [19], leaving women’s experiences and the meanings they assign to those experiences insufficiently understood. A deeper understanding of what women experience when choosing and undergoing VBAC, and of how the caregiving relationship with a midwife shapes that experience, may provide evidence to help midwives and nurses offer more appropriate and supportive care. Accordingly, this study aimed to explore in depth the birth experiences of women who underwent VBAC through a natural birth approach in collaboration with a midwife, using Colaizzi’s [20] phenomenological method. Phenomenological research is appropriate for describing lived experience and exploring its essential meaning [21]. This methodology is particularly suitable for understanding phenomena whose internal processes remain insufficiently explored, including the experiences of women undergoing VBAC. By deepening understanding of the birth experiences of women who choose and undergo VBAC, this study may help support pregnant women’s delivery choices, improve information provision, and provide midwives and nurses with a basis for understanding women seeking VBAC.

The purpose of this phenomenological study was to explore the birth experiences of women who had a midwife-supported VBAC. The study addressed two questions: (1) What factors influenced women’s decisions to pursue VBAC? and (2) What were women’s lived experiences of midwife-supported VBAC?

## Methods

**Ethics statement:** This study was approved by the Institutional Review Board of Kongju National University (KNU_IRB_2024-093).

### Study design

This qualitative study used Colaizzi’s phenomenological method [20] to explore in depth the birth experiences of women who had a midwife-supported VBAC. The study was reported in accordance with the COREQ (Consolidated Criteria for Reporting Qualitative Research) guidelines (https://www.equator-network.org/reporting-guidelines/coreq/).

### Participant selection

Participants were recruited through purposive sampling in accordance with the aims of qualitative research. Eligible participants were women who had experienced VBAC through a natural birth approach in collaboration with a midwife within the previous 3 months, had a history of cesarean delivery, could communicate in Korean, fully understood the study purpose and procedures, and voluntarily agreed to participate. The inclusion criteria were as follows: (1) a history of one or more previous cesarean deliveries, (2) ability to speak, read, and write in Korean, and (3) full understanding of the study purpose and procedures and voluntary agreement to participate. The exclusion criteria were as follows: (1) a history of a vertical or T-shaped uterine incision; (2) a history of induced abortion or dilatation and curettage, which are risk factors for uterine rupture [21]; (3) maternal or fetal health complications related to a present pregnancy, such as gestational hypertension, miscarriage, or preterm birth; and (4) maternal or fetal health complications unrelated to pregnancy, such as pregestational diabetes, renal disease, or hepatic disease. These inclusion and exclusion criteria were established based on systematic reviews of mental health interventions for pregnant women [21,22] and qualitative studies of women undergoing VBAC [13]. Sample size was determined based on data saturation, as appropriate for qualitative research [23]. After each interview, two researchers independently reviewed the transcripts and compared and discussed the emergent themes. After the seventh participant, no new themes were identified, and existing themes appeared to recur. To confirm saturation, additional interviews were conducted with the eighth and ninth participants. Because these interviews also yielded no new themes and only repeated existing themes, data collection was concluded with a final sample of nine participants.

### Data collection

Data were collected from March 1, 2025, to May 7, 2026. Across three hospitals in Seoul, 15 women had a midwife-supported VBAC through a natural birth approach. Of these women, nine expressed willingness to participate and were enrolled in the study. The primary reason recruitment extended over a year was the structural scarcity stemming from South Korea’s exceptionally low VBAC rate (0.17% in 2024) [6]. Furthermore, the pool of candidates meeting the dual condition of experiencing a VBAC via a midwife-supported natural birth was even more limited. This reflects recurrent difficulties reported in recruiting participants for VBAC-related studies in South Korea. Interviews were conducted virtually using the videoconferencing platform Zoom (Zoom Communications, Inc., San Jose, CA, USA). With participants’ consent, the interviews were recorded, and the audio recordings were transcribed immediately after each interview to create the raw data. The virtual format allowed participants to speak freely from their own homes and enabled recruitment without geographic constraints. In general, one interview was conducted per participant, with a mean duration of approximately 50 minutes. For two participants who required supplementary data collection, additional telephone interviews were conducted, with a mean duration of approximately 10 minutes each. The main interview questions were as follows: “Could you tell me about the reasons for and process behind your decision to pursue VBAC?”; “What was the process of preparing for and experiencing natural birth with a midwife like?”; “What was the most memorable experience during the VBAC birth process?”; and “What was your experience of postpartum recovery and newborn care?” During the interviews, exploratory follow-up questions were used according to each participant’s narrative to elicit more in-depth accounts.

### Data analysis

The interview data were analyzed according to the seven-step phenomenological procedure proposed by Colaizzi [20]. In step 1, the researchers repeatedly listened to the audio recordings of the in-depth interviews, transcribed the interviews verbatim, and identified the overall atmosphere and contextual features of the phenomenon. In step 2, significant statements related to the VBAC birth experience were extracted from the participants’ narratives. In step 3, meaningful sentences and phrases were restated in more general and abstract terms. During this stage, the researchers repeatedly returned to the raw data to confirm that the participants’ intended meanings were accurately captured. In step 4, formulated meanings were derived from the restated statements, categorized into themes, and organized into theme clusters. In step 5, the analyzed data were integrated into an exhaustive description according to the themes. In step 6, the fundamental structure of the phenomenon was described. In step 7, member checking was conducted to validate the final description by asking participants whether the identified fundamental structure was consistent with their actual lived experiences.

### Rigor of the study

To ensure rigor in this qualitative study, credibility, confirmability, dependability, and transferability were addressed according to the evaluation criteria proposed by Guba and Lincoln [24]. First, to establish credibility, all interviews were audio- and video-recorded with participants’ consent and transcribed verbatim without omission to prevent data loss. Member checking was then conducted by contacting three participants by telephone and asking whether the derived themes were consistent with their experiences. Participant 8 was presented with the themes “deprivation of agency in childbirth” and “blocked attachment formation”; Participant 7 was presented with “birth reduced to a medical procedure” and “integrated recovery of the body, motherhood, and self”; and Participant 5 was presented with “choosing VBAC amid conflict of will” and “meticulous and continuous care by the midwife.” Through this process, the researchers confirmed that their interpretations were consistent with the participants’ actual experiences. Second, to establish confirmability, before conducting the study, the researchers documented and continuously examined their preunderstandings and biases regarding VBAC and natural birth. Bracketing was also applied during data collection and analysis to prevent the researchers’ preconceptions from influencing the findings and to reflect the participants’ experiences as faithfully as possible. Because of their clinical experience supporting natural and home birth, the researchers held the prior assumption that attempting VBAC could enhance women’s self-efficacy and autonomy. They continuously examined this assumption to ensure that it did not influence data interpretation. Before the in-depth interviews, the researchers shared their preconceptions in team meetings and wrote reflexivity notes during the interviews to maintain a neutral stance and avoid imposing their own biases. Third, to establish dependability, the researchers directly conducted the full transcription and analysis process. Throughout data collection and analysis, an audit trail was maintained by documenting the transcripts, coding lists, categorization process, and rationale for theme derivation in analytic memos. The final findings and overall analytic process were then reviewed for logical consistency by an expert in nursing and midwifery with extensive experience in qualitative research. Fourth, to establish transferability, the participants’ experiences were described using vivid and specific statements. The researchers provided detailed descriptions of the participants’ sociodemographic and obstetric characteristics, including age, parity, education, and occupation; the clinical context in which VBAC occurred, including previous health problems and midwifery support; and the interview context, including timing, setting, and duration. This level of detail was intended to help readers compare the study findings with their own contexts and judge whether the findings may be transferable to similar situations. The researchers have ongoing experience in clinical practice, education, and research related to pregnancy and childbirth. Researcher 1 has more than 20 years of experience conducting qualitative research, teaching women’s health, and running childbirth education classes. Researcher 2 completed graduate-level coursework in qualitative research methodology, has experience conducting qualitative research and presenting findings at academic conferences, and has more than 20 years of experience as a midwife. This clinical background provided Researcher 2 with an in-depth understanding of VBAC experiences and collaborative caregiving relationships, preparing her to explore participants’ experiences in depth.

## Results

### Participant characteristics

Nine married women aged 30–42 years participated (mean age, 37.9 years). One participant (11.1%) had a graduate degree, five (55.6%) had a bachelor’s degree, and three (33.3%) had a high school education or less. Three participants were homemakers; the remaining participants were a teacher, an office worker, a freelancer, a translator/interpreter, a designer, and an English instructor. All had a previous cesarean delivery: eight had undergone one and one had undergone two. Two participants reported health conditions during a previous pregnancy: hypertension during the final month of pregnancy and surgery for septic arthritis at 6 months of pregnancy (Table 1).

### Participants’ vaginal birth after cesarean experiences

Analysis of the interviews with nine participants yielded three categories, 12 theme clusters, and 36 themes. The categories were “Trauma and lasting impressions of cesarean delivery,” “Motives and barriers affecting VBAC decisions,” and “Supportive relationships and transformation of the birth experience” (Table 2).

### Category 1: Trauma and lasting impressions of cesarean delivery

All participants described their previous cesarean delivery not merely as a different mode of birth but as a profound psychological wound. This category represented the starting point of their decision to pursue VBAC and the basis for questioning the meaning of their birth experiences.

#### Theme cluster 1. Birth experience deprived of agency

During their cesarean deliveries, participants felt unable to participate actively in their own births and instead experienced themselves as objects being processed by the medical system. They were excluded from decision-making, procedures were performed without adequate explanation, and the cold, mechanical atmosphere of the operating room was remembered as a space devoid of human warmth. Participants also repeatedly described being deprived of autonomy during surgery: their preferences were ignored, they were not given a choice regarding the method of anesthesia, and the timing of delivery was sometimes determined according to the convenience of clinical staff.

“*Getting on the operating table to deliver my first child did not feel like being treated as a human being; it felt like being treated like a piece of meat. Playing music while administering anesthesia was routine for them, but for me it was such a precious moment. The gap between those two realities was enormous.”* (Participant 8)

“*As soon as they decided to perform surgery, from that moment on, I just felt like I was plopped onto a conveyor belt and pushed along.”* (Participant 7)

“*The operating table was freezing, the lights were glaringly bright, and I just thought to myself that the whole thing was profoundly inhumane.”* (Participant 1)

“*Because the doctor had Wednesday off, the babies had to come out on Tuesday; otherwise, another doctor would deliver them. They asked if I was okay with that... They practically pushed me into it.”* (Participant 3)

“*The hospital where I had my cesarean was actually a good hospital, and I was grateful for many things... those warm words. But ultimately, because I hadn’t studied enough and lacked confidence in myself, I was swayed by those words, which resulted in my decision to undergo surgery.”* (Participant 5)

“*Compared to my first birth, the hospital atmosphere for my second was a bit better, but still, the experience of getting onto the operating table itself was very unpleasant for me.”* (Participant 8)

#### Theme cluster 2. Lack of early maternal-newborn bonding

A common experience among participants was the inability to have immediate physical contact with their newborns because of postoperative physical limitations. This led to missed opportunities for early mother–infant attachment and early breastfeeding. Participants vividly recalled the helplessness of wanting to see their babies but being physically unable to move, as well as the sense of loss they felt when only the baby’s foot was briefly shown before the infant was taken away. Some participants received no guidance on feeding colostrum and therefore did not attempt breastfeeding until 3–4 days after birth, whereas others reported nipple confusion after formula feeding. These experiences contributed to breastfeeding difficulties and diminished the overall quality of the maternal experience.

“*I was separated from my baby the moment the surgery was over. Even though I wanted to go see my baby, I physically couldn’t get there myself, and it was the same for my second.”* (Participant 8)

“*I carried this immense guilt, thinking that if I had just prepared a little more for childbirth, I might not have needed a cesarean section.”* (Participant 9)

#### Theme cluster 3. Pain of long-term physical recovery and a decline in quality of life

Participants reported that recovery from their previous cesarean deliveries lasted from several months to several years. They described burning pain at the incision site, long-term pulling sensations caused by adhesions, the onset of autoimmune disease, and difficulty performing basic activities of daily living, such as sitting up and washing their hair. This severe physical suffering was directly linked to postpartum depressive symptoms and difficulty fulfilling maternal roles.

“*Where they cut me open hurt so badly. It hurt so much that I almost felt resentful toward my baby.”* (Participant 6)

“*The pain buster didn’t work at all... I could feel exactly where they cut, stitched, and tied it tight. That burning sensation. Tears would just stream down my face even when I was lying completely still.”* (Participant 7)

“*After childbirth, my entire body became very weak, and I ended up suffering tremendously from an autoimmune disease for 2 years.”* (Participant 1)

### Category 2: Motives and barriers affecting vaginal birth after cesarean decisions

The decision to attempt VBAC was not driven by a single motive. Participants described a complex psychological process shaped by painful memories of previous cesarean delivery, a deep desire for a different subsequent birth, and social and medical barriers.

#### Theme cluster 1. Inner desire to rewrite the previous childbirth narrative

At the core of the decision to pursue VBAC was a strong internal motivation to recover from the loss and alienation experienced during the previous cesarean delivery. Participants commonly expressed a desire to be the agent of their own birth and to experience the process of giving birth themselves, even if it was painful. For some participants, this motivation was combined with religious values, health considerations related to future pregnancies, and observations of differences in recovery after vaginal and cesarean birth, creating a multifaceted motivational structure.

“*I had this thought that I wanted to rewrite my childbirth experience entirely, so I was determined to take it all the way to the end.”* (Participant 7)

“*I thought to myself, if we ever think about having a second child, I absolutely want to be the true agent of my own childbirth.”* (Participant 7)

“*Because there had been a long interval, I figured I might actually succeed if I tried a VBAC. Also, for religious reasons, I believed that natural childbirth was in accordance with providence. I strongly wanted to try so that I would not have any regrets.”* (Participant 8)

#### Theme cluster 2. Ongoing conflict between will and anxiety

Although participants were strongly committed to VBAC, their resolve wavered repeatedly throughout pregnancy. Anxiety about factors beyond their control that could make VBAC impossible, such as breech presentation, fetal macrosomia, or pregnancy complications, and fear of uterine rupture were the main sources of internal conflict. Participants managed this conflict in their own ways.

“*Because I already knew about VBAC, I decided that if a problem arose, I would absolutely do a VBAC. But you know, I keep second-guessing myself. What if it doesn’t work out?”* (Participant 5)

“*The medical staff literally asked me, ‘Why are you risking your baby’s life like this? Are you confident you won’t regret this later?’ Those words completely haunted me; I couldn’t get them out of my head.”* (Participant 3)

#### Theme cluster 3. Double barriers created by healthcare systems and social biases

When participants expressed a desire for VBAC, most healthcare institutions responded with rejection or active discouragement. Firm opposition from clinical staff, emphasis on the risk of uterine rupture, and the scarcity of VBAC-capable facilities turned the choice of VBAC into a major test of will. Social prejudice also strongly affected participants. Most family members, friends, and acquaintances responded with comments such as “Why go that far?” or “It’s too dangerous,” and even friends who were nurses sometimes opposed the decision. In response, participants used active coping strategies, including sharing information, expressing flexibility, and selectively withholding information.

“*There were almost no hospitals in Busan that would accept VBAC and even the one that did would only do it with induced labor. My biggest fear was that there would be no medical staff to support me.”* (Participant 8)

“*They told me absolutely not. They literally said, ‘Why are you risking your baby’s life like this? Are you confident you won’t regret it later?’”* (Participant 3)

#### Theme cluster 4. Transforming fear through evidence-based information

Participants’ vague fear of uterine rupture was transformed into a more manageable sense of reassurance as they directly reviewed concrete statistical data and academic evidence. ACOG guidelines, criteria related to uterine wall thickness, and VBAC success-rate data played important roles in this process.

“*It turned out the percentage for uterine rupture wasn’t nearly as high as I had feared. Once I saw the actual data, I immediately put those worries to rest.”* (Participant 5)

“*My husband and I did our own homework, constantly looking up research papers from the US and checking what the actual success rates were.”* (Participant 3)

### Category 3: Supportive relationships and transformation of the birth experience

Successful VBAC was not achieved through the woman’s will alone. Rather, the coordinated functioning of a supportive ecosystem—including the spouse, family, midwife, doula, and physician—formed the practical foundation for success. The VBAC experience also carried deep meaning for participants because it allowed them to overcome the loss they had experienced in previous childbirth and reclaim autonomy.

#### Theme cluster 1. Spouse and family support as prerequisites for success

Spousal support was a central resource that sustained participants’ decisions to pursue VBAC. The spouse’s consistent encouragement without coercion, willingness to persuade opposing family members, and presence during birth were decisive forms of support. Conversely, when the spouse strongly opposed VBAC, the mother’s burden of overcoming these barriers through her own will increased.

“*My husband supported me the most. If a mother wants to do a VBAC but her husband says no, I honestly think it would be incredibly hard to succeed.”* (Participant 8)

“*Because my husband knew best how much regret and difficulty I had experienced with our first child, I think he was even more supportive.”* (Participant 7)

“*For people who don’t really understand this, the biggest difference they see is... the cost. So, it can look like we’re just making a fuss over nothing...”* (Participant 5)

#### Theme cluster 2. Professional and continuous midwifery care leading to success

Participants identified the midwife’s role as the most important factor in VBAC success. Midwives supported the women’s active participation through detailed care beginning before 30 weeks of gestation, provision of resources and education, proactive contact at appropriate times, and real-time updates on labor progress. This contrasted sharply with the fragmented, “come and go” communication participants had experienced in standard hospital settings.

“*Because the care (counseling, education) started before 30 weeks, it helped me prepare a lot... It felt as if the midwife provided much more detailed care.”* (Participant 4)

“*If I were to pick the absolute biggest reason I was able to succeed with my VBAC this time, it was definitely the midwife.”* (Participant 5)

“*Because the midwives kept updating me on the progress, I thought, ‘I’m moving forward, I’m doing this right,’ and that helped me endure the pain.”* (Participant 5)

“*I am absolutely 100% certain that if it hadn’t been for my midwife and doula this time, I would never have succeeded in my VBAC.”* (Participant 9)

#### Theme cluster 3. Positive birthing environment and birth autonomy transforming the experience

During the VBAC birth process, participants experienced an environment that differed qualitatively from their previous cesarean deliveries. An environment filled with encouraging language such as “You can do it” rather than “You cannot,” an atmosphere that allowed women to freely change positions, a birth-room structure that allowed family members to be present, and the practice of seeking the woman’s consent even for procedures such as episiotomy contrasted sharply with their previous experiences.

“*No one ever said, ‘You can’t do that.’ In a normal hospital, the first thing they tell you is that if something goes wrong, you need surgery. But here, they told me I could do it, and other mothers next door had just given birth that day, so they only shared positive stories with me.”* (Participant 8)

“*They kept trying to find what was most comfortable for me, saying, ‘Would you like to try this?’ or ‘Try that.’ Even in the middle of the pain, I felt very grateful.”* (Participant 8)

“*They asked me, ‘How would you feel about making a small episiotomy?’ If it meant the baby could come out faster, I was completely willing. But the fact that there was a process of asking me first... I was very grateful for that.”* (Participant 8)

“*What was so good about natural birth was that I could do it together with my first child... We could all be there together, experience it together, and welcome the baby together.”* (Participant 9)

#### Theme cluster 4. Vaginal birth after cesarean success: Restoring agency and completing self-efficacy

At the moment the baby was born, the first emotion participants described was not only joy for the baby but also an intense sense of accomplishment: “I did it.” This was interpreted as an expression of deep self-recovery after overcoming the alienation and loss they had experienced during previous cesarean deliveries. Participants described this experience as the “greatest achievement of my life” and reported that this sense of accomplishment became a source of self-efficacy in parenting and daily life.

“*I realized that I had fully experienced the process of delivering my child, not through surgery, but by actually giving birth. During my cesarean, I felt completely left out of my own delivery, but this time, I gave birth entirely on my own. I truly felt that I had accomplished something together with this child.”* (Participant 8)

“*The sense of accomplishment was so immense that I wondered if I had ever achieved anything this huge in my entire life. Because of that, all the pain was forgotten, and it feels like I’m using that as a driving force, making parenting feel much smoother.”* (Participant 4)

“*I actually gave birth. I even gave birth, what in the world can’t I do? I did not have those thoughts with my first.”* (Participant 7)

#### Theme cluster 5. Rapid physical and emotional recovery and improved maternal role performance after vaginal birth

Physical recovery after VBAC contrasted sharply with recovery after participants’ previous cesarean deliveries. Participants commonly described being able to stand and walk on the day of birth, initiate breastfeeding within 1 hour after birth, and avoid the surgical-site pain that had lasted for months after cesarean delivery. Postpartum depressive symptoms also markedly decreased or disappeared, contributing to improved maternal role performance.

“*After having a natural birth, it was so great that I didn’t have to strain my stomach and could do everything by myself. Someone who had surgery can’t even see their baby on the first day, but with natural birth, I could get right up and do everything from day 1.”* (Participant 8)

“*The reason I’m so glad I chose natural childbirth is that the recovery was, in my standard, unbelievably fast. The speed at which my stomach went down, my uterus contracted, and my milk came in was remarkably faster. I was exclusively breastfeeding by day 4.”* (Participant 1)

“*With my first, I think it was about 21 days when I left the postpartum care center, and I was so depressed up until then... But with my second, I was completely recovered. I didn’t feel depressed at all.”* (Participant 3)

### Fundamental structure of the phenomenon

Women who had experienced cesarean delivery were excluded from decision-making during childbirth, underwent procedures without sufficient explanation, and remembered the cold, mechanical atmosphere of the operating room as a space devoid of human warmth. Although childbirth should have been a “precious moment,” they experienced separation from their newborns, prolonged physical recovery, and deprivation of agency in birth. These experiences created an internal desire to “rewrite the childbirth experience” and became the starting point for their determination to pursue VBAC with the conviction, “Next time, I will give birth myself.” However, this resolve was repeatedly tested by external constraints, including rejection of VBAC by obstetric care providers, fear of uterine rupture, and social prejudice. In this process, women encountered a natural birth environment in collaboration with midwives and developed confidence in their decision through continuous care, evidence-based information, and counseling that respected their values and choices. A humane birth environment not centered solely on medical procedures, the continuous presence of spouses and family members, and women’s active participation in decision-making throughout labor transformed birth from an experience of control and compliance into one of participation and agency. Through the support of spouses and family members, professional and continuous midwifery care that reassured them that they were “on the right track,” and the acquisition of evidence-based information, women built a supportive ecosystem that helped them overcome barriers. During birth, respect for autonomy, encouraging language, and family presence transformed childbirth from alienation into participation. Ultimately, the sense of accomplishment expressed as “I did it” restored the agency lost in previous childbirth, and this achievement, together with rapid physical and emotional recovery, became a source of self-efficacy across motherhood. Thus, VBAC was not simply a change in mode of birth but a holistic process in which women confronted the medical system and social norms, rewrote their birth narratives, and experienced recovery and growth.

## Discussion

This study explored the essential meaning of the birth experiences of nine women who had midwife-supported VBAC through a natural birth approach, using Colaizzi’s [20] phenomenological method. In the category “Trauma and lasting impressions of the cesarean experience,” participants recalled their previous cesarean deliveries as births reduced to medical procedures, which became the basis of their motivation to pursue VBAC. These findings are consistent with a meta-ethnographic study showing that women perceived VBAC as “the journey from pain to power,” through which they overcame suffering related to cesarean delivery and regained strength [13]. Notably, participants consistently reported systematic deprivation of autonomy during surgery, including having their preferences ignored and having the timing of birth determined by clinicians’ personal schedules. This suggests that women’s agency and right to choose during childbirth may be constrained by structural power relationships within the medical system, echoing recommendations emphasizing the need for respectful maternity care (RMC) [8] and previous research on RMC provided by midwives [25]. The findings also suggest that South Korea’s high cesarean delivery rate, which exceeds 60% [6], may not be attributable solely to medical necessity but may also reflect these structural features. Postoperative mother–infant separation and prolonged physical recovery negatively affected maternal role performance and breastfeeding. This finding is consistent with a systematic review of the short- and long-term effects of cesarean delivery, which reported reduced breastfeeding and delayed mother–infant bonding [4]. Similarly, previous meta-analyses have shown that physical pain after cesarean delivery broadly affects health-related quality of life, including walking ability, daily activities, social life, and personal hygiene [26]. The present finding that this pain was linked to postpartum depressive symptoms and diminished quality of parenting experience illustrates the practical reasons women may seek to avoid repeat cesarean delivery.

In the category “Motives and barriers affecting VBAC decisions,” participants’ decision to pursue VBAC reflected a strong internal motivation to rewrite their previous birth narrative. This finding is consistent with a systematic review identifying the desire for vaginal birth, enhanced mother–infant bonding, and expectation of rapid recovery as major facilitators of VBAC choice [15]. As previous studies have reported that prior birth experience, sense of control, and family support have complex effects on VBAC decisions [16], participants in this study also showed a multilayered motivational structure shaped by personal, religious, and physical considerations. However, their strong commitment to VBAC wavered repeatedly throughout pregnancy. Fear of uterine rupture and anxiety about possible failure due to factors beyond their control, such as breech presentation or fetal macrosomia, were major sources of conflict. As reflected in the description that women make VBAC decisions while “groping through the fog” [14], insufficient information and uncertainty can create ongoing psychological conflict. In particular, participants’ experiences of clinicians applying emotional pressure through comments such as “Are you sure you will not regret this?” threatened maternal self-determination, which is consistent with previous findings identifying coercive counseling by healthcare providers as a major barrier to VBAC [15]. South Korea’s very low VBAC attempt rate of less than 1% [6] may be interpreted as reflecting dual barriers created by the healthcare system and social prejudice. Participants found it extremely difficult to locate hospitals that would support VBAC and repeatedly experienced rejection and risk exaggeration from clinicians. Similarly, in the United States, fear of liability has been identified as a major reason obstetricians and midwives avoid VBAC, and cesarean delivery may be chosen for convenience, creating barriers despite women’s preferences [27]. In a United States study of 1,151 VBAC participants, women reported having to fight for VBAC while searching for supportive clinicians, traveling long distances, and overcoming obstacles [28]. The International Federation of Gynecology and Obstetrics (FIGO) recommends that women attempting VBAC give birth in a setting where emergency cesarean delivery is immediately available, with continuous fetal monitoring and care by skilled healthcare professionals [29]. FIGO also emphasizes that women and healthcare professionals should determine the mode of birth through shared decision-making that considers individual risk factors, values, and preferences, supported by balanced information about the benefits and risks of VBAC and repeat cesarean delivery [30]. In this study, participants used diverse coping strategies, including proactive information seeking and flexible attitudes, reflecting a high level of agency and information use among women choosing VBAC.

One of the most prominent findings in the category “Supportive relationships and transformation of the birth experience” was that professional and continuous midwifery care was perceived as a key factor in VBAC success. All participants identified the midwife’s role as the most important single factor in their VBAC success, consistent with findings that continuity of midwifery care improves the quality of the VBAC experience [19]. In particular, individualized education beginning before 30 weeks of gestation, guidance on weight and exercise management, and real-time updates on labor progress provided a foundation for women to participate actively as agents in the birth process. This finding is also consistent with systematic review evidence that midwife-led continuity-of-care models reduce unnecessary medical interventions, improve birth satisfaction, and support mother–infant bonding [13]. It is also consistent with previous research showing that women’s self-advocacy is important for VBAC success and that support from RMC providers, doulas, peers, supportive clinicians, and social media platforms such as Facebook (Meta Platforms, Inc., Menlo Park, CA, USA) can be helpful [28].

Spousal support also functioned as a prerequisite for successful VBAC. Participants perceived that attempting VBAC would be difficult if their spouse opposed it, and they reported that spouses’ empathic understanding and continuous encouragement were central to maintaining their resolve during conflict. This finding is consistent with previous evidence that spouse and family support are major facilitators of VBAC choice [15]. In South Korea, family-centered birth experiences have been identified as the most important determinant of childbirth-care quality, followed by professional support and information provision [30]. Participants in this study recognized that spousal support alone was insufficient and that professionals’ knowledge-based support had unique value that spousal support could not replace. During the VBAC birth process, participants experienced an autonomous birthing environment that differed qualitatively from their previous cesarean experiences. Practices such as freedom to choose positions, encouraging language, family presence, and consent before procedures shifted childbirth from “alienation” to “active participation” [30]. Immediately after birth, participants experienced a sense of accomplishment—“I did it”—even before joy for the baby. This represented not merely the success of a mode of birth but the restoration of agency and self-efficacy that had been diminished in previous births. The finding that this experience translated into self-efficacy in parenting and daily life suggests that the benefits of VBAC are not limited to clinical outcomes such as shorter hospital stays and increased breastfeeding [10], but may extend to women’s psychosocial recovery and formation of maternal identity. Rapid physical and emotional recovery after vaginal birth was reported by all participants. Same-day walking, breastfeeding within 1 hour after birth, and reduced postpartum depressive symptoms were perceived as especially striking in contrast to previous cesarean experiences [31]. These findings suggest that VBAC may affect not only short-term physical recovery but also maternal identity formation and early parenting adaptation, highlighting the need for further research on how mode of birth affects women’s postpartum mental health.

This phenomenological qualitative study helps address a gap in South Korean VBAC research by exploring in depth the experiences of women who underwent VBAC with midwives. Whereas previous studies have focused primarily on clinical indicators such as success rates and uterine rupture risk, this study uses women’s own voices to show that VBAC is more than a choice of birth mode; it is a process of rewriting the birth narrative and achieving self-recovery. These findings provide clinical evidence that may help midwives and nurses caring for women pursuing VBAC better understand their psychosocial experiences. This study has several limitations. First, participants were limited to women who used hospitals specializing in midwife-led natural birth; therefore, their experiences may differ from those of women who undergo VBAC in standard medical settings, limiting the transferability of the findings. Second, although virtual interviews using Zoom allowed participants to be recruited without geographic constraints, this method limited observation of nonverbal expressions that may have been captured in face-to-face interviews. Third, although the small sample size of nine participants was consistent with the principle of saturation in qualitative research, the study may not fully capture the experiences of women from diverse socioeconomic backgrounds. Future research should include expanded qualitative studies of women who experience VBAC in diverse medical settings and contexts, as well as quantitative studies measuring the effects of midwifery support.

The VBAC experience explored in this study shows that VBAC is not simply a change in mode of birth but a holistic process of restoring agency and self-efficacy lost during previous childbirth. First, previous cesarean experiences were remembered as births deprived of agency, marked by mother–infant separation and prolonged physical recovery, and left deep psychological wounds that became the starting point for the decision to pursue VBAC. This indicates that cesarean delivery is not merely a surgical procedure but an event that may have lasting effects on the quality of women’s birth experiences and maternal identity. Second, although the decision to pursue VBAC was driven by an internal desire to rewrite the previous birth narrative, women’s resolve was repeatedly tested by the dual barriers of rejection by the healthcare system and social prejudice. This study suggests that the extremely low rate of attempted VBAC in South Korea reflects not a lack of individual will but a structural problem stemming from the absence of supportive medical environments and institutional foundations. Third, professional and continuous midwifery support was a key factor in VBAC success. Continuous care from early pregnancy through birth, evidence-based education, and real-time information during labor provided a foundation for women to participate actively as agents in childbirth. This underscores the urgent need to expand the role of midwives and establish systems to support VBAC. Fourth, successful VBAC led to an intense sense of self-efficacy—“I did it”—as well as rapid physical and emotional recovery, breastfeeding success, and resolution of postpartum depressive symptoms, standing in sharp contrast to previous cesarean experiences. These findings show that the benefits of VBAC extend beyond maternal physical recovery to maternal role performance and psychosocial well-being.

Based on these findings, the following recommendations are proposed. In clinical practice, midwives and nurses should sensitively recognize the psychological wounds and diminished self-efficacy that women may experience after cesarean delivery and should provide continuous, supportive care to women attempting VBAC from early pregnancy. Respect for women’s autonomy, adequate information provision, and family participation during birth are essential elements of a positive birth experience. In education, midwifery and nursing curricula should include content on the psychosocial experiences of women pursuing VBAC, and evidence-based counseling competencies regarding the safety and benefits of VBAC should be strengthened. At the system and policy levels, policies are needed to expand healthcare institutions that support VBAC and to provide institutional support for midwife-led continuity-of-care models. To reduce South Korea’s high cesarean delivery rate, an environment must first be created in which women can autonomously choose their mode of birth based on sufficient information. In research, expanded qualitative studies including women who have experienced VBAC in diverse medical settings and quantitative studies measuring the effects of midwifery support are needed. Through the lived voices of women who experienced VBAC, this study showed that midwife-supported natural birth is not merely a choice of birth mode but a process of restoring birth agency and achieving holistic growth. These findings may help shift South Korea’s birth culture toward a more woman-centered approach and provide a basis for supportive care practices for women pursuing VBAC.

## Figures and Tables

**Table 1.** Participant characteristics (n=9)

No.	Age (year)	Postpartum interval (reported month/day)	Occupation	Education	Parity	Health conditions in previous pregnancy
1	40	1/14	Translator/interpreter	College	2	Septic arthritis surgery
2	34	2/22	Freelancer	High school	2	None
3	30	2/17	English instructor	College	2	None
4	34	1/14	Office worker	Graduate	2	None
5	40	1/0	Designer	High school	2	None
6	35	2/29	Homemaker	High school	2	None
7	37	1/16	Teacher	College	2	Hypertension
8	39	2/21	Homemaker	College	3	None
9	42	2/21	Homemaker	College	2	None

**Table 2.** Categories, theme clusters, and themes in participants’ experiences of VBAC

Categories	Theme clusters	Themes
Trauma and lasting impressions of the cesarean experience	Birth experience deprived of agency	• Birth reduced to a medical procedure; erosion of agency; compromised dignity; deprivation of bodily control
	Lack of early mother–infant bonding	• Forced separation immediately after cesarean delivery; blocked attachment formation; lost opportunities for early breastfeeding
	Pain of long-term physical recovery and decreased quality of life	• Delayed recovery; delayed attachment; depressive symptoms; impaired maternal role performance and daily life
Motives and barriers affecting VBAC decisions	Inner desire to rewrite the previous birth narrative	• Experience of pain and loss; strong internal motivation to “give birth myself next time”
	Ongoing conflict between will and anxiety	• Firm determination to pursue VBAC; wavering in response to fear of uterine rupture and healthcare-system barriers
	Double barriers created by healthcare systems and social biases	• Repeated rejection by hospitals; negative perceptions of VBAC; VBAC choice as a test of will
	Transforming fear through evidence-based information	• Direct review of data and research findings; transformation of vague fear into concrete reassurance
Supportive relationships and transformation of the birth experience	Spouse and family support as prerequisites for success	• Noncoercive empathic support from the spouse; support for the decision; family acceptance; psychological protection of the mother
	Professional and continuous midwifery care leading to success	• Detailed and continuous midwifery care; real-time guidance from midwives; key professional resources for VBAC success
	Positive birthing environment and birth autonomy transforming the experience	• Encouraging language; freedom to choose positions; family-friendly environment; shift from alienation to active participation in birth
	VBAC success: restoring agency and strengthening self-efficacy	• Sense of accomplishment expressed as “I did it”; overcoming loss from previous childbirth; reintegration of body, motherhood, and self
	Rapid physical and emotional recovery and improved maternal role performance after vaginal birth	• Rapid recovery; successful breastfeeding; reduced postpartum depressive symptoms; contrast with cesarean delivery; strengthened maternal role performance

VBAC: Vaginal birth after cesarean.
